# Trait impulsivity and acute stress interact to influence choice and decision speed during multi-stage decision-making

**DOI:** 10.1038/s41598-020-64540-0

**Published:** 2020-05-08

**Authors:** Candace M. Raio, Anna B. Konova, A. Ross Otto

**Affiliations:** 1 Neuroscience Institute, New York University Grossman School of Medicine, New York, USA; 2 Department of Psychiatry, Rutgers University - New Brunswick, Piscataway, USA; 3 Department of Psychology, McGill University, Montreal, Canada

**Keywords:** Psychology, Human behaviour

## Abstract

Impulsivity and stress exposure are two factors that are associated with changes in reward-related behavior in ways that are relevant to both healthy and maladaptive decision-making. Nonetheless, little empirical work has examined the possible independent and joint effects of these factors upon reward learning. Here, we sought to examine how trait impulsivity and acute stress exposure affect participants’ choice behavior and decision speed in a two-stage sequential reinforcement-learning task. We found that more impulsive participants were more likely to repeat second-stage choices after previous reward, irrespective of stress condition. Exposure to stress, on the other hand, was associated with an increased tendency to repeat second-stage choices independent of whether these choices previously led to a reward, and this tendency was exacerbated in more impulsive individuals. Such interaction effects between stress and impulsivity were also found on decision speed. Stress and impulsivity levels interacted to drive faster choices overall (again irrespective of reward) at both task stages, while reward received on the previous trial slowed subsequent first-stage choices, particularly among impulsive individuals under stress. Collectively, our results reveal novel, largely interactive effects of trait impulsivity and stress exposure and suggest that stress may reveal individual differences in decision-making tied to impulsivity that are not readily apparent in the absence of stress.

## Introduction

A prominent question that has emerged across animal and human models of decision-making concerns how individual differences shape the way in which reinforcement drives subsequent choice behavior. The propensity to repeat previously rewarded actions and avoid those that do not yield reward is a fundamental tenet of decision-making^1^ and highlights the critical role that learning plays in the choices we make. In recent years, this tightly coupled relationship has been investigated using reinforcement learning (RL) approaches that formalize how the value of candidate actions are learned through experienced outcomes and how distinct valuation systems may contribute to decision control^2–4^. In addition to providing a mechanistic understanding of how one’s history of reinforcement drives subsequent choice behavior, these computational approaches also afford the opportunity to examine how individual differences shape distinct features of reward-driven behavior^5–9^.

A growing body of work supports the notion that both trait-like individual differences and more transient changes in affective state exert observable effects on an individual’s responsivity to rewarding outcomes^10,11^—and, further, how these outcomes simultaneously shape subsequent choices and the speed with which these choices are made. Two such prominent trait- and state-like factors that have garnered considerable attention in the literature are impulsivity and stress exposure, respectively. Impulsivity—a multidimension construct defined broadly as the general disposition to rash action, including, but not limited to, acting without substantial forethought or consideration of potential consequences, waiting/motor impulsivity and perseverance, and inattention^12–15^—has been shown to relate to poor financial, health, social and professional outcomes^16–18^, and to contribute significantly to almost all psychiatric disorders marked by pathological choice, such as substance use^19–24^ and impulse control disorders^25^, obesity^26–29^, and excessive gambling^30^. The observation that impulsive behavior typically emerges in rewarding contexts suggests that an individual’s trait level of impulsivity may be associated with differences in reward responsivity^31,32^. Accordingly, a growing body of research has aimed to more formally characterize the role that impulsivity might play in reward-related learning and choice behavior^33–40^.

Similarly, a growing literature points to a potent effect of acute stress exposure upon multiple reward-related functions, including valuation, learning and choice implementation^41^. Stressors are defined as real or perceived threats that trigger a cascade of neurophysiological responses that include rapid autonomic nervous system activity followed by systemic release of glucocorticoids^42–44^. The marked cognitive and physiological responses engendered by stressors are well positioned to modulate behavioral responses to reward. For example, acute stress has been shown to increase reward responsivity^45,46^, diminish the capacity to flexibly update value in dynamic learning environments^41,47,48^ and shift decision control away from more complex, model-based choice learning strategies^49,50^. These findings yield support for stress exposure playing a role in dysfunctional or maladaptive reward-seeking behavior, as seen in disorders such as addiction^51,52^ and depression^53,54^.

Although both stress and impulsivity have played prominently in the decision-making literature, little work has examined the interaction between these constructs in the context of RL paradigms permitting assessment of sequential effects on features of choice behavior. This interaction is especially relevant to understand given that stress and impulsivity are often thought to alter reward learning in similar ways. For example, both acutely stressed participants and participants high in trait impulsivity exhibit increased response repetition after positive reinforcement, and faster response times (RTs) in simple RL tasks^16,45–47^. These effects on learning and decision-making are thought to occur either by stress ‘occupying’ or impairing cognitive resources that allow for more deliberative decision-making processes^55,56^ or by both factors imposing internally perceived time constraints that manifest in differential choice speeding^25,57,58^. However, it remains unclear whether stress and impulsivity similarly and independently influence features of reward learning, or whether they interact such that stress acts jointly with trait impulsivity to alter choice behavior.

**Figure 1:**
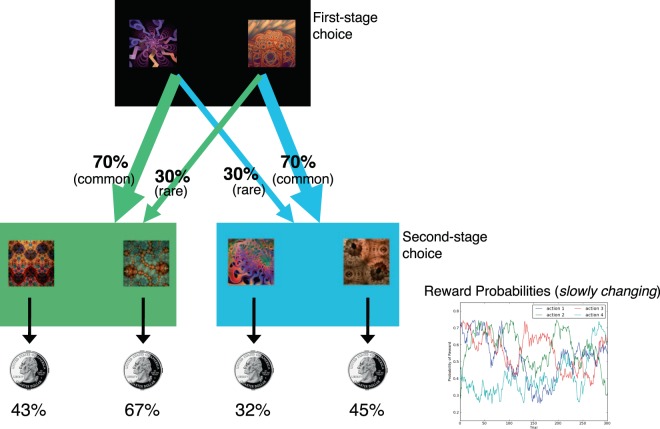
Structure of the Two-Stage reinforcement-learning task. In each trial, subjects chose between two initial options, leading to either of two second-stage choices (green or blue states), for different, slowly changing, chances of monetary reward. Each first-stage option preferentially led to one of the two second-stage states (“common”), however, on 30% of trials (“rare”) it instead led to the other.

Accordingly, the present study sought to examine this possible interaction between acute stress and trait impulsivity, focusing on choice and decision speed in the context of a simple sequential decision-making task. To do so, we leveraged an existing data set originally collected to test how acute stress affects the relative expression of model-based and model-free learning in the “two-step task”^59^ (see Fig. 1). These two reward-learning strategies are thought to reflect separate valuation systems, with ‘model-based’ control taking into account the broader structure of the environment to plan actions in a deliberate manner, and ‘model-free’ control promoting computationally ‘cheaper’ but more reflexive, habitual behavior. In a previous investigation, we found that stress selectively reduced model-based control, seemingly sparing model-free learning^49^, a now established finding in the literature^60,61^. Yet, few studies (including our previously published work) have taken full advantage of the inherent structure of the two-step task—which involves making sequential choices that allow for a more refined examination of an individual’s responsivity to previous rewarding and/or surprising events (e.g., state transitions). For example, an individual’s second-stage choice behavior (see detailed task description below) aligns closer to simple probabilistic reward learning, which acute stress is documented to alter^46,62^, while first-state choices allow for examination of how immediately preceding rewards impact choices—and RTs—that never lead to immediate rewards. Here, we take advantage of the sequential nature of this task to probe how both impulsivity and stress affect two features of reward learning: the probability of choosing rewarded options and choice speed (RT), at each choice stage.

Based on prior findings using simple RL tasks, we hypothesized that impulsivity and acute stress might exert independent or joint effects on learning as indicative of enhanced reward responsivity. Specifically, we expect these factors will be associated with an increased propensity to repeat rewarded choices (and perhaps choice more generally, irrespective of reward, given purported deficits in deliberative decision-making processes), and that this may selectively emerge in second-stage choices, which—unlike first-stage choices—can yield immediate rewards. Given previous work on impulsivity and RT^63^, as well as past theoretical accounts that stress intensifies implicit (or internally-imposed) time pressure^57^, we further expect impulsivity and stress to affect choice RTs. While we have reason to believe these constructs should affect RTs similarly across both choice stages, it is possible that we may see divergence in RTs among first-stage choices that can uniquely occur after previous reward.

## Methods

### Participants

Fifty-six healthy individuals participated in the study (30 female, age: *M* = 25.67 years; *SD* = 7.27 years) and were paid 5 cents per rewarded trial to incentivize performance. The proportions of females in control and stress conditions were 0.50 and 0.58 respectively (see below). All research and experimental procedures were approved by the New York University Committee on Activities Involving Human Subjects and were performed in accordance with these approved Institutional Review Board guidelines and regulations. Written informed consent was obtained from all participants. Following our earlier study which utilized this dataset^49^, we identified and excluded participants who failed to meet a response deadline on more than 15 trials (n = 3), and who failed to demonstrate responsivity to reward as defined by repeating previously rewarded second-stage responses on less than 50% of trials (n = 4).

### Impulsivity assessment

Participants were administered the BIS-11 questionnaire as a measure of trait impulsivity^64^, which consists of 30 statements, such as “I do things without thinking” and “I am more interested in the present than the future” with which participants stated their level of agreement on a four-point scale. Higher summed scores indicate higher levels of impulsivity. Total BIS-11 scores ranged from 33 to 88 (*M* = 58.0, *SD* = 10.44). Importantly, BIS-11 scores did not differ significantly between the control (*M* = 58.15, *SD* = 11.36) and stress conditions (*M* = 57.80, *SD* = 9.06) [*t* = 0.11, *p* = 0.91], described below.

### Acute stress manipulation

Participants were randomly assigned to undergo a stress or control manipulation prior to the task. In the stress condition (n = 20), participants underwent the Cold Presser Task (CPT)^65^, during which they were asked to immerse their right hand up to and including the wrist for 3 min in ice water (0–5 °C). Participants in the control condition (n = 28) submerged their right hand up to and including the wrist for 3 min into room temperature water (21–30 °C). Immediately after the manipulation, participants indicated on a scale ranging from 0 (“not at all”) to 10 (“very much”) how unpleasant they found the immersion procedure. As reported in our original study, the CPT manipulation successfully evoked a subjective stress response: participants in the stress condition reported that the CPT was significantly more unpleasant (*M* = 6.68, *SD* = 0.54) than those in the control condition (*M* = 2.19, *SD* = 0.38) [*t* = 6.95, *p* < 0.001].

To assess physiological stress responses, saliva samples were also collected throughout to assess participants’ cortisol levels, using an absorbent oral swab that participants placed under their tongues for 2 min. To control for diurnal rhythms in cortisol levels, all participants were run between 1 pm and 6 pm. Sample collection occurred at baseline after a 10 min acclimation period (s1), immediately after baseline cognitive measures (not reported) and task instructions (s2, ~25 min after s1), 10 min after CPT administration (s3, ~43 min after s1), and immediately following the task (s4, ~64 min after s1). Samples were frozen and preserved immediately after collection at −30 °C and transported frozen to a CLIA-certified analytical laboratory where cortisol concentrations were determined with high-sensitivity enzyme immunoassay kits (Salimetrics, LLC, State College, PA). Duplicate assays were conducted for each sample interval, and the average of the two values was used in our analyses.

Cortisol responses were found to peak during the task (i.e, 10 min after the stress manipulation, cf. Figure 2 of Ref. ^49^). As previously reported, we found a significant interaction between condition (stress/control) and time of cortisol measurement (*F* = 19.99, *p* < 0.0001), such that only participants in the stress group exhibited a marked increase in cortisol response. Within both stress and control groups, cortisol concentrations did not change significantly between s3 and s4 (*p*s > 0.54) further suggesting that cortisol concentrations remained stable throughout the RL task. Thus, to facilitate interpretability of potential interaction effects with impulsivity, our analyses of stress effects focused on condition assignment rather than participant-level cortisol response.

**Figure 2:**
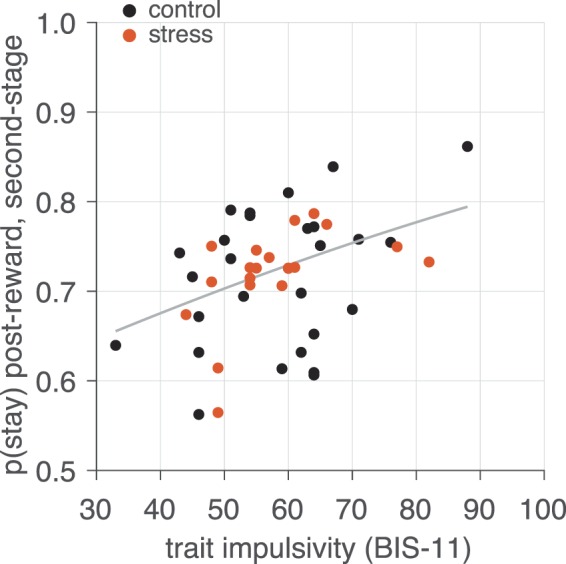
Visualization of the effect of previous reward upon second-stage choice stay probability, as a function of impulsivity level (BIS-11 score). The individual effects plotted are the estimated per-subject logistic regression coefficients from the group analysis (conditioned on the group-level estimates) superimposed on the estimated group-level effect. The regression line is computed from the group-level (fixed-effect) estimated from the logistic regression (Table 1).

### Two-step decision-making task

Participants performed 200 trials of the two-step RL task^59^ (Fig. 1), originally employed in our prior study to dissociate parameterized indices of model-free and model-based strategic contributions to choice behavior. In each two-stage trial, participants first made a choice between two options (depicted as fractals; first-stage), which probabilistically lead to one of two second-stage “states” (colored green or blue). In each of these subsequent states, subjects made another choice between two options (second-stage choice), which were associated with different probabilities of monetary reward. Choosing one of the first-stage options led to one of the second-stage states most of the time (70%) and led to the other second-stage state the remaining 30% of the time. Because the second-stage reward probabilities independently change over time, decision-makers need to make trial-by-trial adjustments to their choices in order to effectively maximize payoffs.

Prior to initiating the task, participants were provided with task instructions and completed 10 practice trials to familiarize themselves with the task structure and response procedure. Note that at this point, the control and stress groups were subject to identical procedures and thus differences in choice behavior cannot be attributed to the conditions under which task instructions were received. Following administration of the stress/control manipulation and cortisol sample s3, participants completed 200 trials of the two-step RL task (Fig. 1).

In the first stage, two fractal images appeared on a black background (indicating the initial state), and there was a 1.5 s response window during which participants could choose the left- or right-hand response using the “Z” or “?” keys, respectively. After a response was entered, the selected option was highlighted for the rest of the response window. The background color also changed in accordance with the second-stage state. After this transition, participants’ chosen first-stage action moved to the top of the screen. Two fractal images, corresponding to the actions available in the second-stage, were displayed; participants had 1.5 s to make a response. As in first-stage choice, the selected option was highlighted for the rest of the response window. Then, either a picture of a coin (indicating that they had been rewarded on that trial) or the number zero (indicating that they had not been rewarded that trial) was shown. The probability of receiving a reward for either second-stage choice was determined by an independent drifting Gaussian random walk (SD = 0.025) with reflecting boundaries at 0.25 and 0.75. The mapping of actions to stimuli and transition probabilities were randomized across participants.

### Data analysis

Our analysis approach relied upon a mixed-effects regression approach similar to that used to analyze choice behavior in previous studies using this task^59,66^. Because in our current report we examined first-stage and second-stage choices separately, our regression models jointly analyzed behavior across the two stages using dummy variables specifying at which stage each effect is estimated (see Appendix for syntax used to specify the models). This also allowed us to separately estimate ‘baseline’ repetition rates and RTs at each stage (effectively allowing for a separate intercept term at each choice stage). At the first stage we estimated the trial-by-trial effect of the previous trial’s reward and at the second stage we estimated the trial-by-trial effect of previous reward (conditioned upon the last visit to that second-stage state) and the transition type (common versus rare) that led to that second-stage state. These models were estimated using the lme4 package for the R programming language^67^.

In the model examining choices, we specified a mixed-effects logistic regression (using *glmer* in R) to simultaneously explain the first-stage choice on each trial (coded as stay versus switch relative to the last first-stage choice made) and the second-stage choice (coded as stay versus switch relative to the last second-stage choice made in that particular state). A second mixed-effects regression model (using *lmer* in R) was specified for RTs. RTs were log-transformed to remove skewness and RTs exceeding 3 *SD*s from a participant’s mean RT were excluded from analysis in both the choice- and RT-predicting models^68^, resulting in 17,974 total observations. To account for practice effects in regressions predicting RTs, a linear predictor of trial number was additionally included. In both the choice and RT models, within-subject factors were taken as random effects across subjects, and parameter estimates and statistics reported are at the population level. Continuous covariates (BIS-11 scores and trial numbers) were entered into the regressions as z-scores. All other binary predictor variables were coded −1/1 in the case of the choice model and coded 0/1 in the case of the RT model.

Significance testing of individual regression coefficients was performed using Satterthwaite’s degrees of freedom method implemented in the lmerTest package^69,70^ in the case of the RT-predicting model and Wald tests, as implemented by the lme4 package, in the case of the choice-predicting model. This multilevel modeling approach yields conservative parameter estimates that preclude the need to adjust for multiple comparisons^71^. Because of the partial pooling inherent in multilevel models, coefficient estimates are “shrunk” toward a population-level mean, effectively correcting for the increased risk of false positives incurred by testing all effects of interest within a single model^72^.

## Results

Our previous study examined how acute stress affected the expression of model-based and model-free learning at the first stage as described in detail in Otto, Raio, *et al*. (2013). We note that since our originally reported effects of stress on model-based learning are reported in this previous work, and others’, they are not considered further in the current manuscript. Here, our analysis focused on possible relationships between acute stress and individual differences in impulsivity, and their interaction, on first- and second-stage choice behavior as a function of previous rewards—in effect, focusing on ‘model-free’ features of choice—and choice RTs as a function of previous rewards and state transitions.

### First-stage choice behavior

As is typically observed in two-stage tasks of this kind, we found a significant main effect of previous reward on first-stage choice behavior such that participants were more likely to repeat previously rewarded first-stage actions (*β*_*stage 1*× *previous reward*_ term_,_ *p* < 0.0001; see Table 1 for full regression coefficient estimates and degrees of freedom). This main effect of previous reward is usually interpreted as the contribution of a ‘model-free’ RL strategy^49,59,66^. This first-stage responsivity to previous rewards did not appear to be influenced by either stress condition, impulsivity level, or their interaction (*β*_*stage 1*× *previous reward* × *stress*_ term, *p* = 0.910, *β*_*stage 1*× *previous reward* × *BIS-11*_ term, *p* = 0.463, *β*_*stage 1*× *previous reward* × *stress* × *BIS-11*_ term, *p* = 0.892, respectively). In addition, there were no significant main or interaction effects on participants’ tendency to choose the same option overall irrespective of previous reward (all *p* > 0.428; see Table 1).

**Table 1:** Mixed-effects logistic regression coefficients indicating the influence of trial-to-trial variables, impulsivity level, and stress condition upon choice repetition.

Coefficient	Estimate (SE)	df	p-value	Coefficient	Estimate (SE)	df	p-value
stage 1	1.8423 (0.162)	1	<0.0001*	stage 2	0.8222 (0.0784)	1	<0.0001*
stage 1 × stress	0.0203 (0.1669)	1	0.903	stage 2 × stress	0.1793 (0.0801)	1	0.025*
stage 1 × BIS-11	0.0818 (0.1742)	1	0.639	stage 2 × BIS-11	0.1005 (0.0842)	1	0.233
stage 1 × stress × BIS-11	0.138 (0.1741)	1	0.428	stage 2 × stress × BIS-11	0.2692 (0.0842)	1	0.001*
stage 1 × previous reward	0.8838 (0.0811)	1	<0.0001*	stage 2 × previous reward in state	0.9258 (0.0568)	1	<0.0001*
stage 1 × previous reward × stress	−0.0093 (0.0819)	1	0.91	stage 2 × previous reward in state × stress	−0.0275 (0.0579)	1	0.635
stage 1 × previous reward × BIS-11	−0.0636 (0.0865)	1	0.463	stage 2 × previous reward in state × BIS-11	0.1437 (0.0614)	1	0.019*
stage 1 × previous reward × stress × BIS-11	−0.0117 (0.0865)	1	0.892	stage 2 × previous reward in state × stress × BIS-11	0.0117 (0.0615)	1	0.85
stage 1 × previous transition × stress	0.0217 (0.0381)	1	0.569	stage 2 × current transition	0.1939 (0.0451)	1	<0.0001*
stage 1 × previous transition × BIS-11	0.0613 (0.0435)	1	0.158	stage 2 × current transition × stress	0.0858 (0.0452)	1	0.058
stage 1 × previous transition × stress × BIS-11	0.0087 (0.0431)	1	0.84	stage 2 × current transition × BIS-11	0.0084 (0.0481)	1	0.862
				stage 2 × current transition × stress × BIS-11	−0.0137 (0.0482)	1	0.776

Asterisks denote significance at the 0.05 level.

### Second-stage choice behavior

We next examined second-stage choice (which could lead to immediate reward), conditioned upon choice made on the participant’s last visit to that second-stage state and as a function of reward obtained on that visit. We found a significant main effect of reward (*β*_*stage 2* × *previous reward in state*,_ *p* < 0.0001; Table 1), mirroring previous findings^73^. This effect of previous reward conditioned on the last visit to that state is indicative of a ‘win-stay’ strategy and can also be taken as an index of responsivity to recent reward feedback^46^.

**Figure 3:**
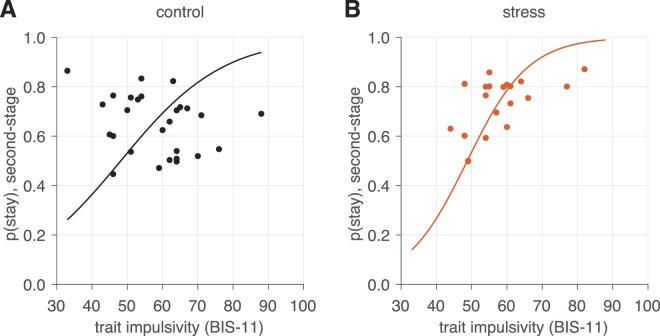
Visualization of the effect impulsivity level upon overall rate of second-stage choice stay probability, in the (**A**) control and (**B**) stress conditions. Individual effects plotted are the estimated per-subject logistic regression coefficients from the group analysis (conditioned on the group-level estimates) superimposed on the estimated group-level effect. The regression line is computed from the group-level (fixed-effect) estimated from the logistic regression (Table 1).

Importantly, we found this win-stay like behavior increased with individuals’ level of impulsivity (Fig. 2), as indicated by a significant interaction between recent reward feedback at the second-stage and impulsivity level (*β*_*stage 2*× *previous reward in state* × *BIS-11*_ term, *p* = 0.019; Table 1). However, the relationship between impulsivity level and responsivity to recent rewards did not appear to depend on participants’ assigned stress condition (*β*_*stage 2*× *previous reward in state* × *BIS-11* × *stress*_ term, *p* = 0.850; Table 1). Instead, the overall tendency to repeat second-stage choices (that is, regardless of whether they were previously rewarded or not) was predicted by stress condition (*β*_*stage 2* × *stress*_ term, *p* = 0.025; Table 1), an effect qualified by a stress condition by impulsivity level interaction (Fig. 3; *β*_*stage 2* × *BIS-11* × *stress*_ term, *p* = 0.001). That is, stressed participants exhibited a greater tendency to select the same options regardless if they previously led to reward, and this effect was specifically present in more impulsive participants under stress. These results indicate that repeating previously rewarded choices at the second-stage was related to impulsivity—but not stress condition—while choice repetition in general was predicted by stress condition and its interactive effect with impulsivity.

### First-stage RTs

**Figure 4:**
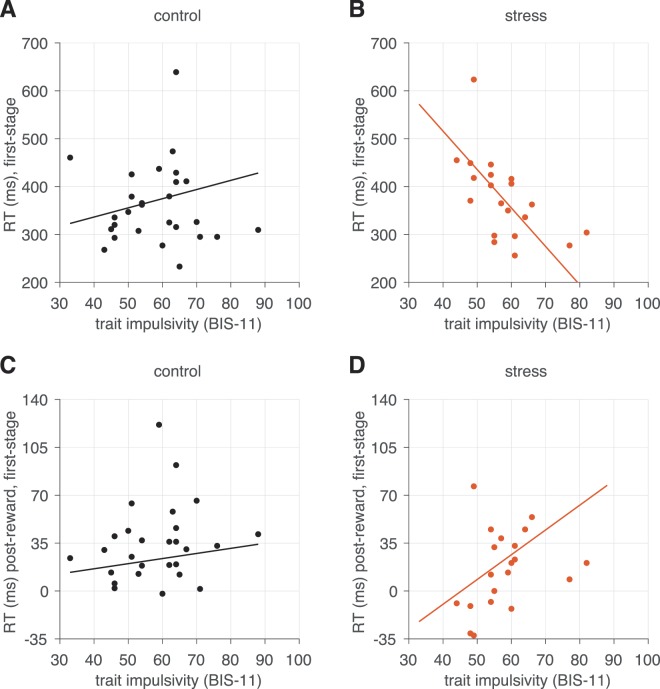
Visualization of the effect of impulsivity level upon overall first-stage RTs in the (**A**) control and (**B**) stress conditions, and upon first-stage differential RTs following previous reward (versus non-reward) in the (**C**) control and (**D**) stress conditions. Median first-stage choice RTs (or post-reward - no-reward RT differences) for individual subjects are depicted on the vertical axis. The regression line is computed from the group-level (fixed-effect) estimated from the mixed regression model (Table 2).

We next examined whether first-stage choice RTs—irrespective of recent reward outcomes—differed by impulsivity level and acute stress. Neither impulsivity (*β*_*stage 1* × *BIS-11*_ term, *p* = 0.891) nor stress condition (*β*_*stage 1* × *stress*_ term, *p* = 0.215) alone predicted overall first-stage RTs (see Table 2 for full regression coefficient estimates). However, we observed a negative interaction between stress and impulsivity level, such that response speeding increased in more impulsive individuals under stress (Fig. 4A,B; *β*_*stage 1* × *stress* × *BIS-11*_ term, *p* = 0.003). In other words, acute stress selectively increased choice speed in more impulsive participants, while this relationship with impulsivity was not present under control conditions.

**Table 2:** Mixed-effects regression coefficients indicating the influence of trial-to-trial variables, impulsivity level, and stress condition upon log-transformed RTs.

Coefficient	Estimate (SE)	df	p-value	Coefficient	Estimate (SE)	df	p-value
stage 1	5.8301 (0.035)	46.55	<0.0001*	stage 2	6.1987 (0.0413)	48.05	<0.0001*
stage 1 × stress	0.067 (0.0532)	43.19	0.215	stage 2 × stress	0.0245 (0.0648)	43.64	0.707
stage 1 × BIS-11	−0.0044 (0.0316)	43.13	0.891	stage 2 × BIS-11	0.0143 (0.0389)	44.09	0.715
stage 1 × stress × BIS-11	−0.1729 (0.0559)	43.06	0.003*	stage 2 × stress × BIS-11	−0.1525 (0.0688)	44.04	0.032*
stage 1 × key rep	0.0219 (0.0085)	79.05	0.012*	stage 2 × key rep	0.0003 (0.0114)	45.26	0.982
stage 1 × previous reward	0.0811 (0.013)	46.71	<0.0001*	stage 2 × previous reward in state	−0.016 (0.0126)	44.09	0.212
stage 1 × previous reward × stress	−0.032 (0.0198)	43.75	0.114	stage 2 × previous reward in state × stress	−0.0267 (0.0192)	42.86	0.172
stage 1 × previous reward × BIS-11	0.0076 (0.0117)	43.89	0.52	stage 2 × previous reward in state × BIS-11	−0.0138 (0.0114)	43.69	0.231
stage 1 × previous reward × stress × BIS-11	0.0477 (0.0209)	45.44	0.027*	stage 2 × previous reward in state × stress × BIS-11	0.0384 (0.0203)	45.12	0.065
stage 1 × previous transition × stress	0.0095 (0.0137)	318.50	0.492	stage 2 × current transition	0.0754 (0.0197)	42.22	<0.001*
stage 1 × previous transition × BIS-11	−0.0001 (0.0105)	601.00	0.991	stage 2 × current transition × stress	0.0813 (0.0303)	42.33	0.01*
stage 1 × previous transition × stress × BIS-11	−0.0073 (0.0188)	642.70	0.698	stage 2 × current transition × BIS-11	0.0209 (0.0178)	42.03	0.246
				stage 2 × current transition × stress × BIS-11	−0.0156 (0.0315)	42.56	0.623

Asterisks denote significance at the 0.05 level.

We also observed a tendency for a reward received on the previous trial to slow subsequent first-stage RTs (*β*_*stage 1* × *previous reward*_ term, *p* < 0.0001). This post-reward tendency for slowing was significantly predicted by an interaction between impulsivity level and stress condition (*β*_*stage 1 previous reward* × *stress* × *BIS-11*_ term, *p* = 0.027) (Fig. 4C,D). This effect was not significant for impulsivity (*β*_*stage 1* × *previous reward* × *BIS-11*_ term, *p* = 0.52) nor stress condition (*β*_*stage 1* × *previous reward* × *stress*_ term, *p* = 0.114) alone, suggesting that slowing after previous reward is observed only when more impulsive participants were stressed.

### Second-stage RTs

**Figure 5:**
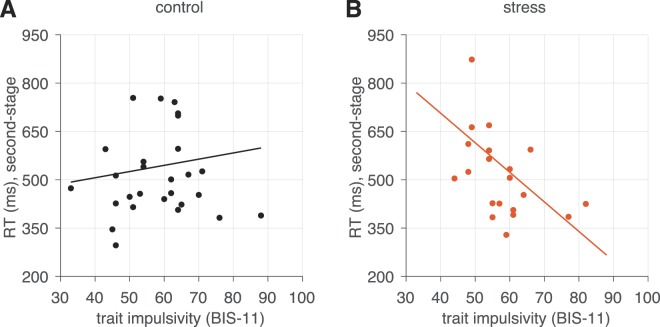
Visualization of the effect of impulsivity level upon second-stage RTs in the (**A**) control and (**B**) stress conditions. Median second-stage choice RTs for individual subjects are depicted on the vertical axis. The regression line is computed from the group-level (fixed effect) estimated from the mixed regression model (Table 2).

A similar pattern of results emerged for RTs associated with second-stage choices. Choice speed at the second-stage (irrespective of previous reward) was not affected by impulsivity or assigned stress condition (*β*_*stage 2* × *BIS-11*_ term, *p* = 0.715 and *β*_*stage 2* × *stress*_ term, *p* = 0.707, respectively). However, again, the interaction between stress condition and impulsivity level on RTs was significant (*β*_*stage 2* × *stress* × *BIS-11*_ term, *p* = 0.032), suggesting that only stressed participants who are more impulsive made faster choices overall at the second-stage (Fig. 5).

Unlike the first-stage choice RTs, we did not find that reward received on the immediately preceding trial in that state slowed subsequent second-stage RTs made in the same state (*β*_*stage 2* × *previous reward in state*_ term, *p* = 0.212), nor did we find these RTs to be predicted by impulsivity level (*β*_*stage 2* × *previous reward in state* × *BIS-11*_ term, *p* = 0.231). However, we did find that previous reward in the same state tended to slow subsequent second-stage RTs in more impulsive participants under stress, although this interaction between impulsivity level and stress condition was only observed at trend level significance (*β*_*stage 2* × *previous reward in state* × *stress* × *BIS-11*_ term, *p* = 0.065).

### Effect of transition structure

**Figure 6:**
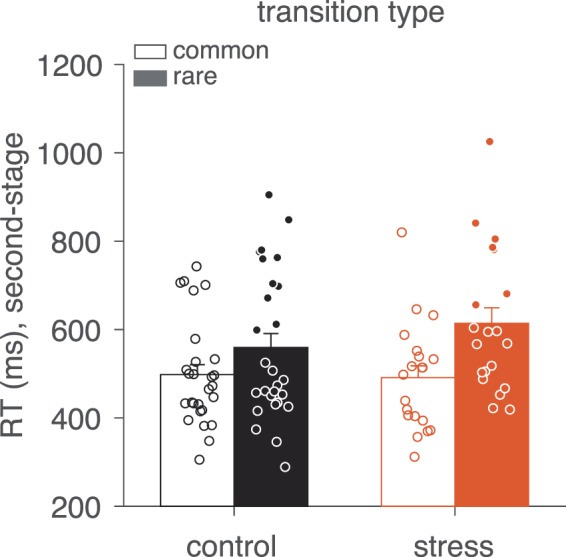
Median second-stage choice RT as a function of current transition type (common versus rare) and stress condition (stress versus control). Scatter points represent individual subject median RTs. Error bars represent standard error of the mean (SEM).

As previous work with the same task has found that second-stage RTs are slower following rare transitions than common transitions^66,73^, we also examined how this second-stage slowing was related to participants’ impulsivity level and stress. Post-transition slowing is thought to reflect surprise resulting from an uncommon event (assuming participants have knowledge of the task transition structure). We found that, as previously observed, participants’ second-stage choices were slower following rare transitions than following common transitions (Fig. 6; *β*_*stage 2* × *current transition*_ term, *p* < 0.001) but interestingly, this slowing effect was exacerbated by acute stress such that participants in the stress condition exhibited a larger slowing effect than control participants (*β*_*stage 2* × *stress* × *current transition*_ term_,_ *p* = 0.01, see Table 2, Fig. 6).

## Discussion

Impulsivity and exposure to acute stress are two prominent factors thought to alter reward-related learning and decision-making, yet few investigations have examined how these factors might jointly alter RL processes in humans. Leveraging a two-stage RL task, we examined how reward-contingent (and surprise-contingent) choice and decision speed are shaped by acute stress exposure and trait impulsivity.

Based on the extant literature, we hypothesized that impulsivity and acute stress might independently—or jointly—exert effects on learning that would be indicative of enhanced reward responsivity. Indeed, we observed that the tendency to repeat a previously reinforced choice increased with higher impulsivity levels, suggesting a “win-stay” or “Law of Effect” tendency prevails in more impulsive individuals. Importantly, this relationship between impulsivity and reward responsivity only emerged in second-stage choice behavior where choices result in *immediate rewards*, but not in first-stage choices that cannot lead directly to reward. This is consistent with a broader conceptualization of trait impulsivity as exerting a stronger effect on behavior in immediately rewarding contexts^32^. Interestingly, while impulsivity levels predicted this manifestation of reward responsivity, acute stress exposure alone yielded no such effect, nor did it interact with impulsivity to influence this behavior. This suggests that impulsivity appears to selectively render individuals more susceptible to repeat rewarded (but not unrewarded) choices, a dissociation that was revealed because an inherent feature of our task structure is that second-stage choices are immediately followed by an outcome while first-stage choices are not.

We did, however, observe a main effect of stress condition as well as an interaction between acute stress and impulsivity in overall choice tendencies, such that more impulsive participants under stress were more likely to repeat second-stage choices regardless of whether these choices were previously rewarded. This perseveration-like behavior is consistent with notions that stress and impulsivity impair deliberative decision processes^45–50^ and suggest these factors may interact to render participants less sensitive or precise in their representation of the task’s reward structure, such that choices are repeated even if only a subset of them are rewarded. The finding that this interaction was significant only in second-stage choices is consistent with the fact that only second-stage choices are associated with immediate rewards, while first-stage choices are not. Together, these results suggest that higher impulsivity levels increase the propensity to repeat previously rewarded actions (akin here to better learning), but that this propensity generalizes to unreinforced choices when coupled with stress exposure. This points to stress exposure as driving a potentially maladaptive form of choice perseveration or repetition—especially in more impulsive individuals—that persists independent of reward outcome. This inflexible adjustment of choice behavior to reinforcement has also been documented in previous investigations showing that acute stress exposure leads to reduced responsivity to both positive and negative feedback during the learning phase of probabilistic reward tasks^45,46^.

Not only did acute stress render impulsive participants more likely to repeat choices independent of reward, but it also made choices faster in a reward-independent manner, particularly in the first-stage. Specifically, under stress, more impulsive participants made faster choices overall, while this effect was not present under control conditions. The fact that stress increases decision speed in more impulsive participants is consistent with theoretical^57^ and empirical^58^ accounts that suggest stress might engender ‘internal’ time pressure, which may already be a trait-like feature of impulsive individuals’ decision process^63^, that is most pronounced under stress.

While impulsive participants were faster to respond when under stress, a distinct RT profile emerged for responses following reward. Specifically, we found that receiving a reward on the previous trial (i.e., at the second-stage) *slowed* subsequent first-stage RTs. This post-reinforcement slowing was more prevalent among impulsive individuals when under acute stress. Such post-reinforcement “pausing” has been described in a broad range of reward-based choice tasks across species as slower RTs after reward receipt or ‘wins’^74–78^ as well as faster response rates after unexpected omission of reward or loss^34,36,78,79^. This post-reward slowing is generally thought to reflect greater attentional or orienting responses to receipt of reward. However, one possibility is that rewards tend to incur reward prediction errors (PEs)^59^, which has been found to slow subsequent choices in a variety of tasks^80,81^. While we do not measure PEs directly, theoretically, an enhanced responsivity to PEs in stressed individuals who are more impulsive could potentially arise from changes in expectation of reward, which is consistent with the neuromodulatory changes imposed by stress exposure, particularly dopamine—which drives PE signaling^59,82^ and changes rapidly after stress exposure^56,82^. Post-reinforcement pausing may thus result from elevated orienting response to reward receipt in impulsive individuals under stress. An alternate possibility is that after reward receipt, participants experience greater conflict before making subsequent first-stage choices (given the nature of our two-stage task), an account supported by recent demonstrations of RT slowing with greater decision difficulty or conflict^83,84^. Such decision conflict may be exacerbated in impulsive individuals under stress, consistent with the well-established finding that stress decreases cognitive capacity and flexibility. Future work using joint modeling approaches—such as recent demonstrations using RL models paired with drift diffusion models^83–85^—could help clarify the interplay between these choice and RTs effects.

Although the overall patterns of RTs observed at the second-stage mirrored that of the first-stage for the interactive effect of impulsivity and stress on overall RTs—i.e., regardless of previous reward, more impulsive subjects were faster under stress—we observed a distinct pattern between the two stages with respect to RTs following a previously rewarded choice. Specifically, unlike first-stage RTs, reward-based slowing of second-stage RTs was not modulated jointly by impulsivity and stress. This divergence may arise from differences in reward availability in each choice stage stemming from the task structure (Fig. 1). Second-stage choices can lead to immediate reward, while first-stage choices cannot. Consequently, only first-stage choices can immediately follow the receipt of reward. Thus, our ability to detect an effect of acute stress and impulsivity upon choice and RTs following feedback might require an immediately preceding rewarding event. Future model-based work examining RTs in sequential RL tasks, following recent work by Shahar and colleagues^85^, will be especially important to better understand how individual differences drive such differential effects on choice behavior at distinct stages.

Finally, consistent with previous work, we found that second-stage RTs were slower following rare transitions than common transitions^66,86^, and further, that, while not related to impulsivity, this slowing effect was exacerbated by stress. This responsivity to transition frequency has been interpreted as a reflection of knowledge of the transition structure, wherein uncommon transitions engender surprise (i.e., expectancy violation^66^). Here, acute stress—but not trait impulsivity—appeared to intensify this response to surprising events, suggesting that acute stress could tune individuals to simple violations of expectations. Interestingly, recent work identifies surprise as a key driver of (subjective) stress response^87^, suggesting the possibility of a bidirectional, positive feedback relationship between surprise stemming from the environment and the acute stress response.

Notably, the sequential structure of the choice task enabled us to probe simple relationships between trait impulsivity and acute stress and how choice and RT are affected by previous reinforcement in a way that could not be revealed in a standard RL task. Specifically, the sequential nature of the task allowed us to examine the serial effect of choice (e.g. how reward received following second-stage choices affected subsequent first-stage choices), which would not be possible to ascertain without this two-stage feature. This task structure further afforded the opportunity to independently characterize how choice behavior and decision speed changed in first- vs. second-stage components of the task as a function of trait impulsivity and stress exposure.

Converging lines of research have highlighted the importance of characterizing how both individual differences and affective state can shape learning and decision-making processes. Our results reveal novel interactive effects of trait impulsivity and stress exposure and suggest that rather than exerting a direct effect on reward repetition and choice speeding, stress appears to reveal choice tendencies in individuals higher in trait-impulsivity. This pattern is corroborated by work that points to stress as a factor that reveals underlying choice biases rather than exerting a purely directional effect on choice behavior^41,88^.

Our findings motivate a number of future research directions. First, while impulsivity is undoubtedly a multidimensional construct^16^, here we used the BIS-11^64^, a widely used and validated self-report measure of impulsive behavior to measure and define impulsivity. Future research may seek to test how more circumscribed forms of impulsivity (negative and/or positive urgency, choice impulsivity, rapid-response or motor impulsivity, etc.) shape choice behavior in RL tasks. Second, here we examined the effect of physiological stress. It is possible that other forms of acute stress exposure (e.g., social stress), long-term stress exposure (e.g., chronic stress, life adversity) or individual differences in trait anxiety may exert distinct effects on choices and RTs in RL tasks, which will be important to examine in future work. Future work should also certainty explore the interaction between stress and impulsivity in larger and more diverse samples in order to fully understand the joint effects of these factors on RL processes. Extending this work using computationally-informed approaches can offer a more detailed account of how impulsivity and stress shape choice behavior, furthering our understanding of how these constructs can give rise to psychological dysfunction.

## Appendix

Syntax used for mixed-effects logistic regression on choice behavior using the glmer() function in the lme4 package for R:

stay ~ 0 + stage 1 + stage 2 + z bis 11 + cond + stage 1:(cond + z bis 11 + last win + z bis 11:cond + z bis 11:(last win) + cond:(last win) + z bis 11:cond:(last win)) + stage 2:(cond + z bis 11 + last win in state + z bis 11:cond + z bis 11:(last win in state) + cond:(last win in state) + z bis 11:cond:(last win in state)) + (0 + stage 1 + stage 2 + stage 1:last win + stage 2:(last win in state) | subj)

Syntax used for mixed-effects regression on log-transformed choice RTs using the lmer() function in the lme4 package for R:

log_rt ~0 + stage 1 + stage 2 + stage 1:(z bis 11 + cond + z bis 11:cond + last win + z trial num + z bis 11:(last win) + z bis 11:cond:(last win)) + stage 2:(z bis 11 + cond + z bis 11:cond + last win in state + trans + z trial num + z bis 11:(last win in state + trans) + cond:(last win in state + trans) + z bis 11:cond:(last win in state + trans)) + (0 + stage 1 + stage 2 + stage 1:(z bis 11 + cond + last win + z trial num + z bis 11:(last win) + z bis 11:cond:(last win)) + stage 2:(z bis 11 + cond + last win in state + trans + z trial num + z bis 11:(last win in state + trans) + z bis 11:cond:(last win in state + trans))

## References

[1] Thorndike, E. L. *Animal intelligence: Experimental studies*. The Macmillan company (1911).

[2] Frank MJ, Claus ED (2006). Anatomy of a decision: Striato-orbitofrontal interactions in reinforcement learning, decision making, and reversal. Psychological Review.

[3] Daw ND, Niv Y, Dayan P (2005). Uncertainty-based competition between prefrontal and dorsolateral striatal systems for behavioral control. Nat Neurosci..

[4] O’Doherty JP, Cockburn J, Pauli WM (2017). Learning, Reward, and Decision Making. Annual Review of Psychoogy.

[5] Wit S (2012). Corticostriatal Connectivity Underlies Individual Differences in the Balance between Habitual and Goal-Directed Action Control. Journal of Neuroscience.

[6] Flagel SB, Watson SJ, Robinson TE, Akil H (2007). Individual differences in the propensity to approach signals vs goals promote different adaptations in the dopamine system of rats. Psychopharmacology (Berl.)..

[7] Doll BB, Hutchison KE, Frank MJ (2011). Dopaminergic Genes Predict Individual Differences in Susceptibility to Confirmation Bias. Journal of Neuroscience.

[8] Badre D, Doll BB, Long NM, Frank MJ (2012). Rostrolateral prefrontal cortex and individual differences in uncertainty-driven exploration. Neuron.

[9] Patzelt EH, Hartley CA, Gershman SJ (2018). Computational phenotyping: Using models to understand individual differences in personality, development, and mental illness. *Personality*. Neuroscience.

[10] Frank MJ, Moustafa AA, Haughey HM, Curran T, Hutchison KE (2007). Genetic triple dissociation reveals multiple roles for dopamine in reinforcement learning. Proceedings of the National Academy of Sciences.

[11] Phelps EA, Lempert KM, Sokol-Hessner P (2014). Emotion and decision making: Multiple modulatory neural circuits. Annual Review of Neuroscience.

[12] Evenden J (1999). Varieties of impulsivity. Psychopharmacology.

[13] Hamilton KR (2015). Choice impulsivity: Definitions, measurement issues, and clinical implications. Personality Disorders.

[14] Hamilton KR (2015). Rapid-response impulsivity: definitions, measurement issues, and clinical implications. Personality Disorders.

[15] Cyders MA (2015). The misnomer of impulsivity: Commentary on “Choice Impulsivity” and “Rapid-Response Impulsivity” Articles by Hamilton and Colleagues. Personality Disorders.

[16] Bari A, Robbins TW (2013). Inhibition and impulsivity: Behavioral and neural basis of response control. Progress in Neurobiology.

[17] Bechara A (2005). Decision making, impulse control and loss of willpower to resist drugs: a neurocognitive perspective. Nature Neuroscience.

[18] Franken IH, van Strien JW, Nijs I, Muris P (2008). Impulsivity is associated with behavioral decision-making deficits. Psychiatry Research.

[19] Schlagenhauf F (2009). Ventral striatal activation during reward anticipation correlates with impulsivity in alcoholics. Biological Psychiatry.

[20] Bickel WK, Jarmolowicz DP, Mueller ET, Koffarnus MN, Gatchalian KM (2012). Excessive discounting of delayed reinforcers as a trans-disease process contributing to addiction and other disease-related vulnerabilities: emerging evidence. Pharmacological Therapy.

[21] Kale D, Stautz K, Cooper A (2018). Impulsivity related personality traits and cigarette smoking in adults: A meta-analysis using the UPPS-P model of impulsivity and reward sensitivity. Drug and Alcohol Dependence.

[22] Jentsch JD (2015). Dissecting impulsivity and its relationships to drug addictions. Annals of the New York Academy of Sciences.

[23] Amlung M, Vedelago L, Acker J, Balodis I, MacKillop J (2017). Steep delay discounting and addictive behavior: a meta-analysis of continuous associations. Addiction.

[24] Lee B (2009). Striatal dopamine D2/D3 receptor availability is reduced in methamphetamine dependence and is linked to impulsivity. Journal of Neuroscience.

[25] Voon V (2010). Impulsive choice and response in dopamine agonist-related impulse control behaviors. Psychopharmacology (Berl)..

[26] Nederkoorn C, Smulders FT, Havermans RC, Roefs A, Jansen A (2006). Impulsivity in obese women. Appetite.

[27] Schag F, Schonleber J, Teufel M, Zipfel S, Giel KE (2013). Food-related impulsivity in obesity and Binge Eating Disorder – a systematic review. Obesity Reviews.

[28] Jarmolowicz DP (2014). Robust relation between temporal discounting rates and body mass. Appetite.

[29] Meule A, Platte P (2015). Facets of impulsivity interactively predict body fat and binge eating in young women. Appetite.

[30] Ioannidis K, Hook R, Wickham K, Grant JE, Chamberlain SR (2017). Impulsivity in gambling disorder and problem gambling: A meta-analysis. American Journal of Drug and Alcohol Abuse.

[31] Crews FT, Boettiger CA (2009). Impulsivity, frontal lobes and risk for addiction. Pharmacol Biochem Behav.

[32] Dalley JW, Everitt BJ, Robbins TW (2011). Impulsivity, compulsivity, and top-down cognitive control. Neuron.

[33] Kim S, Lee D (2011). Prefrontal cortex and impulsive decision making. Biological Psychiatry.

[34] Gipson CD (2012). A translational behavioral model of mood-based impulsivity: Implications for substance abuse. Drug and Alcohol Dependence.

[35] Wise RJ, Phung AL, Labuschagne I, Stout JC (2014). Differential effects of social stress on laboratory-based decision-making are related to both impulsive personality traits and gender. Cognition and Emotion.

[36] Verbruggen F, Chambers CD, Lawrence NS, McLaren IPL (2017). Winning and losing: Effects on impulsive action. Journal of Experimental Psychology: Human Perception and Performance.

[37] Canale N, Rubaltelli E, Vieno A, Pittarello A, Billieux J (2017). Impulsivity influences betting under stress in laboratory gambling. Scientific Reports.

[38] Shao R, Read J, Behrens TEJ, Rogers RD (2013). Shifts in reinforcement signalling while playing slot-machines as a function of prior experience and impulsivity. Translational Psychiatry.

[39] Cáceres P, San Martín R (2017). Low cognitive impulsivity is associated with better gain and loss learning in a probabilistic decision-making task. Frontiers in Psychology.

[40] Otto AR, Markman AB, Love BC (2012). Taking More, Now: The Optimality of Impulsive Choice Hinges on Environment Structure. Social Psychological and Personality Science.

[41] Porcelli AJ, Delgado MR (2017). Stress and decision-making: effects on valuation, learning, and risk-taking. Current Opinions in Behavior Sciences.

[42] Selye H (1936). A syndrome produced by diverse nocuous agents. Nature.

[43] Ulrich-Lai YM, Herman JP (2009). Neural regulation of endocrine and autonomic stress responses. Nature Reviews Neuroscience.

[44] McEwen BS (2007). Physiology and neurobiology of stress and adaptation: central role of the brain. Physiol Rev.

[45] Mather M, Lighthall NR (2012). Both risk and reward are processed differently in decisions made under stress. Current Directions in Psychological Science.

[46] Lighthall NR, Gorlick MA, Schoeke A, Frank MJ, Mather M (2013). Stress modulates reinforcement learning in younger and older adults. Psychology and Aging.

[47] Petzold A, Plessow F, Goschke T, Kirschbaum C (2010). Stress reduces use of negative feedback in a feedback-based learning task. Behavioral Neuroscience.

[48] Raio CM, Hartley CA, Li J, Orederu T, Phelps EA (2017). Stress exposure attenuates flexible updating of aversive value. Proceedings of the National Academy of Sciences.

[49] Otto AR, Raio CM, Chiang A, Phelps EA, Daw ND (2013). Working-Memory Capacity Protects Model-Based Decision-Making from Stress. Proceedings of the National Academy of Sciences.

[50] Schwabe L, Wolf OT (2011). Stress-induced modulation of instrumental behavior: from goal-directed to habitual control of action. Behavioral Brain Research.

[51] Sinha R (2001). How does stress increase risk of drug abuse and relapse?. Psychopharmacology (Berl).

[52] Koob GF (2013). Addiction is a reward deficit and stress surfeit disorder. Frontiers in Psychiatry.

[53] Hodes GE, Kana V, Menard C, Merad M, Russo SJ (2015). Neuroimmune mechanisms of depression. Nature Neuroscience.

[54] Corral-Frias NS (2015). Stress-related anhedonia is associated with ventral striatum reactivity to reward and transdiagnostic psychiatric symptomatology. Psychological Medicine.

[55] Hermans EJ, Henckens MJ, Joels M, Fernandez G (2014). Dynamic adaptation of large-scale brain networks in response to acute stressors. Trends in Neuroscience.

[56] Arnsten AF (2009). Stress signalling pathways that impair prefrontal cortex structure and function. Nature Reviews Neuroscience.

[57] Hockey G (1997). Compensatory control in the regulation of human performance under stress and high workload: A cognitive-energetical framework. Biological Psychology.

[58] Lenow JK, Constantino SM, Daw ND, Phelps EA (2017). Chronic and acute stress promote overexploitation in serial decision- making. Journal of Neuroscience.

[59] Daw ND, Gershman SJ, Seymour B, Dayan P, Dolan RJ (2011). Model-based influences on humans’ choices and striatal prediction errors. Neuron.

[60] Radenbach C (2015). The interaction of acute and chronic stress impairs model-based behavioral control. Psychoneuroendocrinology.

[61] Park H, Lee D, Chey J (2017). Stress enhances model-free reinforcement learning only after negative outcome. PLOS ONE.

[62] Berghorst LH, Bogdan R, Frank MJ, Pizzagalli DA (2013). Acute stress selectively reduces reward sensitivity. Frontiers in Human Neuroscience.

[63] Wittmann M, Paulus MP (2008). Decision making, impulsivity and time perception. Trends in Cognitive Sciences.

[64] Patton JH, Stanford MS, Barrett ES (1995). Factor structure of the Barratt Impulsiveness Scale. Journal of Clinical Psychology.

[65] McRae AL (2006). Stress reactivity: biological and subjective responses to the cold pressor & Trier Social stressors. Human Psychopharmacology.

[66] Decker JH, Otto AR, Daw ND, Hartley CA (2016). From creatures of habit to goal-directed learners: Tracking the developmental emergence of model-based reinforcement learning. Psychological Science.

[67] Bates, D., & Maechler, M. *lme4: Linear mixed-effects models using S4 classes*. Retrieved from http://CRAN.R-project.org/package=lme4 (2009).

[68] Ratcliff R (1993). Methods for dealing with reaction time outlier. Psychological Bulletin.

[69] Kuznetsova, A., Brockhoff, P. B., & Christensen, R. H. B. lmerTest Package: Tests in Linear Mixed Effects Models. *Journal of Statistical Software*, **82**(13), 10.18637/jss.v082.i13 (2017).

[70] Luke SG (2017). Evaluating significance in linear mixed-effects models in R. Behavioral Research Methods.

[71] Baayen RH, Davidson DJ, Bates DM (2008). Mixed-effects modeling with crossed random effects for subjects and items. Journal of Memory and Language, Special Issue: Emerging Data Analysis.

[72] Bornemann B, Kovacs P, Singer T (2019). Voluntary upregulation of heart rate variability through biofeedback is improved by mental contemplative training. Scientific Reports.

[73] Otto AR, Gershman SJ, Markman AB, Daw ND (2013). The curse of planning: Dissecting multiple reinforcement learning systems by taxing the central executive. Psychological Science.

[74] Peters H, Hunt M, Harper D (2010). An animal model of slot machine gambling: The effect of structural characteristics on response latency and persistence. Journal of Gambling Studies.

[75] Dixon MR, Schreiber JE (2004). Near-miss effects on response latencies and win estimations of slot machine players. The Psychological Record.

[76] Dixon MJ, MacLaren V, Jarick M, Fugelsang JA, Harrigan KA (2013). The frustrating effects of just missing the jackpot: Slot machine near-misses trigger Large skin conductance responses, but no post-reinforcement pauses. Journal of Gambling Studies.

[77] Delfabbro PH, Winefield AH (1999). Poker-machine gambling: An analysis of within session characteristics. British J of Psychology.

[78] Leslie, J. C. Principles of behaviour analysis. Amsterdam: Harwood Academic Publishers (1996).

[79] Wyckmans, F. *et al*. Reduced model-based decision-making in gambling disorder. *Scientific Reports* **9**(1), 10.1038/s41598-019-56161-z (2019). 10.1038/s41598-019-56161-z PMC6927960 31873133

[80] Holroyd CB, Krigolson OE (2007). Reward prediction error signals associated with a modified time estimation task. Psychophysiology.

[81] Montague PR, Dayan P, Sejnowski TJ (1996). A framework for mesencephalic dopamine systems based on predictive Hebbian learning. Journal of Neuroscience.

[82] Butts KA, Weinberg J, Young AH, Phillips AG (2011). Glucocorticoid receptors in the prefrontal cortex regulate stress-evoked dopamine efflux and aspects of executive function. Proceedings of the National Academy of Sciences.

[83] Fontanesi L (2019). A reinforcement learning diffusion decision model for value-based decisions. Psychonomic Bulletin Review.

[84] Pedersen ML, Frank MJ, Biele G (2017). The drift diffusion model as the choice rule in reinforcement learning. Psychon Bull Rev..

[85] Shahar N, Hauser T, Moutoussis M, Moran R, Keramati M (2019). NSPN consortium *et al*. Improving the reliability of model-based decision-making estimates in the two-stage decision task with reaction-times and drift-diffusion modeling. PLoS Comput Biol.

[86] Deserno L (2015). Ventral striatal dopamine reflects behavioral and neural signatures of model-based control during sequential decision making. PNAS.

[87] de Berker AO (2016). Computations of uncertainty mediate acute stress responses in humans. Nature Communications.

[88] Starcke K, Brand M (2016). Effects of stress on decisions under uncertainty: a meta-analysis. Psychologicall Bulletin.

